# Long-term mortality among women with epithelial ovarian cancer: a population-based study in British Columbia, Canada

**DOI:** 10.1186/s12885-018-4970-9

**Published:** 2018-10-25

**Authors:** Nimisha Arora, Aline Talhouk, Jessica N. McAlpine, Michael R. Law, Gillian E. Hanley

**Affiliations:** 1 Department of Obstetrics & Gynecology, University of British Columbia, Vancouver, BC Canada; 2 Centre for Health Services and Policy Research, School of Population and Public Health, University of British Columbia, Vancouver, BC Canada; 3 Diamond Health Care Center, Vancouver General Hospital, 6207A 2775 Laurel St., Vancouver, BC V5Z 1M9 Canada

**Keywords:** Ovarian Cancer, Histotype, Mortality, Survival

## Abstract

**Objectives:**

Among women with epithelial ovarian cancer (EOC), histotype is one of the major prognostic factors. However, few data are available on histotype- specific survival and mortality estimates among these patients. We therefore examined survival and causes of death among women with EOC by histotype.

**Methods:**

A population- based cohort including all ovarian cancer patients diagnosed in British Columbia (BC) between 1990 and 2014 was built using population-based administrative datasets. We compared causes of death within histotypes, by age at diagnosis, *BRCA* status, and time since diagnosis.

**Results:**

A total of 6975 women were identified as having been diagnosed with EOC between 1990 and 2014 in BC. The most common cause of death among these women was ovarian cancer until 10 years post diagnosis when other causes surpassed ovarian cancer as the leading cause of death. Among women with serous EOCs, ovarian cancer was the leading cause of death 12 years after diagnosis, whereas ovarian cancer was the leading cause of death for 8 years among women with non- serous EOCs. Among women with serous EOCs, ovarian cancer was the leading cause of death for 12 years among younger women (< 60 years of age) compared to 8 years among women > = 60 years of age, and those with *BRCA* mutations were more likely to die from ovarian cancer than those without a *BRCA* mutation.

**Conclusions:**

Within 10 years from diagnosis, ovarian cancer is the leading cause of death among women diagnosed with EOC.

**Electronic supplementary material:**

The online version of this article (10.1186/s12885-018-4970-9) contains supplementary material, which is available to authorized users.

## Introduction

Ovarian cancer is the leading cause of death among women with gynecologic cancers. Although lifetime risk of ovarian cancer in the general population is relatively low (1.4%) [1], it is the fifth leading cause of cancer deaths among women in Canada, with a 5-year survival rate of 44% [2] compared with nearly 90% [3]for breast cancer, more than 80% [2] for endometrial cancer, and nearly 73% [2] for cervical cancer. While survival is much improved when ovarian cancer is detected in the early stages, there are presently no effective screening methods demonstrated to reduce mortality [4]. Ovarian cancer is also largely asymptomatic in early stages, thus approximately 70% of women are diagnosed when the disease is already at advanced stages (Stage III and IV) [5].

Causes of death among cancer patients has been relatively well studied across many different forms of cancer. It is often reported that within 5 years of a cancer diagnosis, cancer is the most common cause of death. However, the risk of dying from cancer decreases with time from diagnosis and cancer patients become more likely to die from other causes [5]. This has been reported for breast cancer [6], prostate cancer [7], head and neck cancer [8], and lung cancer [9]. With respect to ovarian cancer, a previous American study using Surveillance Epidemiology, and End Results (SEER) data has reported that the probability of dying from ovarian cancer decreases with time, but that ovarian cancer remains the leading cause of death 15 years post diagnosis among women diagnosed in advanced stages [5].

Here, we have focused on EOC, which represents 90% of all ovarian cancers [10]. Over the past decade, it has become apparent that EOC is a heterogenous disease, comprising of distinct histotypes that differ in presentation, response to therapy, molecular features, hereditary predisposition, site of origin and clinical outcomes. Although EOC histotypes share an anatomical location (the ovary), they are now considered distinct diseases. [11–15]. This histotype-specific approach has completely changed the approach to clinical care and research. Histotype and stage remain the strongest prognostic factors in EOC. Some international work examining survival among ovarian cancer patients by histologic-subtypes has reported that survival rates were lowest among women with high-grade serous cancers [13]. However, little is known about causes of death among women with EOC, and we do not currently have evidence on whether causes of death differ among these affected women by histotype.

Histotype-specific mortality estimates are of clinical importance as information may impact advice given or interventions undertaken for patients and physicians involved in their care. For women with OC histotypes less likely to recur, information is currently lacking on their potential health concerns following EOC diagnosis We therefore examined survival and causes of death among women with EOC in British Columbia between 1990 and 2014 by histotype and years since EOC diagnosis.

## Methods

### Data sources

In this descriptive study, we built a population-based cohort using data from the BC cancer registry [16], Vital statistics [17], BC hereditary cancer program (HCP) [16], and BC’s insurance registry file (the Consolidation file) [18]. The BC Cancer Registry is a population-based registry of all cancers diagnosed in BC residents. It receives notifications of cancer from many sources including pathology, cytology and other labs, hospital charts, death certificates, and admissions to cancer centers operated by the BC Cancer Agency. The Registry contains personal and demographic information and information about the specific cancer diagnosis. The vital statistics death file is an extract of the deaths registration file provided by BC vital statistics agency. It contains information on all deaths in BC, including underlying cause of death (UCOD) and exact date of death. We accessed data from the HCP, the source of all *BRCA1 and BRCA2* testing in the province of BC. We classified patients as having a *BRCA* mutation if they had either a *BRCA1* or *BRCA2* mutation or both. The consolidation file is a comprehensive data set, containing information on individuals receiving health services and/or individuals eligible to receive health services in BC, Canada (˜4.6 million people). We used the consolidation data to access demographic data and information on registration for health insurance in BC, in order to assess whether a woman had moved out of BC [19]. As the data linkage maintained patient anonymity (all identifiers were removed before being provided to the researchers) and the population-based administrative datasets in BC operate based on passive consent (i.e. patients may withdraw their consent and their data will be removed from the administrative datasets), direct patient consent was not required. With the permission of all relevant data stewards, and ethics approval from the University of British Columbia’s behavioural research ethics board, data were retrieved from PopData BC [20]. All inferences, opinions, and conclusions drawn are those of the authors and do not reflect the opinions or policies of the Data Stewards.

### Study cohort

Our study population consists of all patients diagnosed with ovarian cancer in BC between 1990 and 2014. The *International Statistical Classification of Diseases codes*: *tenth revision* (ICD-10) was used to identify these women with ovarian cancer with the codes of C56.0 (ovary), and C57. 0 (fallopian tube) in the BC cancer registry. Following WHO criteria (histotype classification), the study cohort was restricted to patients diagnosed with serous, mucinous, endometrioid, and clear cell tumours. The codes are unable to distinguish between high grade and low-grade serous cancers, and thus we have classified them as serous cancers. There are also ovarian cancers with morphology codes that are not detailed enough to classify into histologic subtypes and were included as “Not classified” histologic subtype. Women with these unclassified tumours are analyzed separately in all histotype-specific analyses. To ensure we had complete follow-up on women included in our cohort and to prevent misclassifying women as alive if they had left the province, we first assessed whether women were registered for health care in BC in the final year of follow-up (2014). If women were not registered in 2014 and were not captured in the death file, we required that they be registered for > 5 years following their EOC diagnosis in order to be included in our study.

### Assessment of causes of death

We classified the UCODs using ICD-10 categories. The UCODs were classified into specific categories such as ovarian cancer, breast cancer, colorectal cancer, ‘other’ cancers (lung cancer, gastrointestinal tract cancer, blood lymph cancer, other malignancy, non malignant and unspecified), cardiovascular diseases (rheumatic, hypertension, ischemic, heart failure, congenital, pulmonary, cardiomyopathy etc*)*, other chronic conditions (diabetes, COPD, AIDS/HIV, pneumonia, other infectious and parasitic disease, asthma, cerebro and other vascular disease, liver disease, pulmonary fibrosis etc), external causes (Motor vehicle accidents, poisoning, falls, suicide, other unintentional injuries etc), and unclassified causes (causes of death that did not meet the criterion of the above categories) (see Table 1). Patients were considered to have died of ovarian cancer if the cause of death was reported as ovarian cancer or cancer-related likely due to ovarian cancer (which included deaths from neoplasm of uterus, cervix, placenta, ovary and adnexa, vagina and external genitalia following an initial diagnosis of ovarian cancer).

**Table 1:** Underlying causes of death and their ICD-10 codes

Causes of Death	ICD-10 codes
Ovarian cancer or ovarian cancer related	C510-C58
Breast cancer	C500-C509
Colorectal cancer	C180-C218
Other cancer	Lung: C33, C340-C349, C384, C450; GI: C150-C179, C220-C269; Blood lymph: C810-C969, C463; Other malignancy: C000-C148, C300-C449, C451-C462, C467-C499, -C609, C620-C768, C5091, C80; Non malignant and unspecified: D000-D489
Cardiovascular	Rheumatic/Valvular: I050-I099, I340-I38; Hypertension: I10-I159; Ischemic: I200-I259; Conductive & Dysrhythmic: I440-I499; heart failure: I500-I509; Congenital: Q200-Q249; Pulmonary: I260-I289; Cardiomyopathy: I420-I429; Unspecified: I312-I318, I510-I513, I515-I519
Other chronic	Diabetes: E100-E149; COPD: J440-J449 AIDS/HIV: B200-B24; Pneumonia: J120-J181, J188-J189 Other infectious and parasitic disease: A000-B199, B250-B999, U049; Asthma: J450-J459, J46; Cerebro and other vascular disease: I600-I698, I700-I879, I950-I959, I880-I899; Liver disease:K700-K7699; Pulmonary fibrosis: J841; ALS/MS: G122, G1221, G35; Lung disease due to external agents: J60-J709
External causes of death	Motor vehicle accidents (MVA): V020-V049, V090-V099, V120-V149, V190-V196, V200-V799, V803-V805, V820-V821, V823-V839, V840-V878, V880-V888, V8900-V8909,V8920-V8929, V8990-V8999, Y850 Poisoning: X40-X49 Falls: W00-W19 Suicide: X60-X84, Y870 Other external:Y10-Y369, Y890-Y899 Other unintentional injuries:, V010, V019, V050-V069, V091, V099, V100-V119, V150-V189, V198-V199, V250-V259, V350-V359, V450-V459, V550-V559, V650-V659, V750-V759, V800-V802, V806-V819, V822, V879, V889, V910-V919, V930-V949, V950-V978, V98-V99, W20-W64, W75-W99, X20-X39, X50-X59, Y40-Y849, Y859, Y86, Y880-Y883
Unclassified cause of death	Codes that did not meet criterion above

### Statistical analysis

Women were monitored as of the date of their ovarian cancer diagnosis (as recorded in the BC cancer registry database) until their death or until December 31st, 2014 (the end of the follow-up period). Causes of death were stratified based on histotype categories (Serous, Mucinous, Endometrioid, Clear cell, Not classified). We further stratified based on age at diagnosis (< 60 or > =60 years) and *BRCA* mutation status. Causes of death were calculated as percentages with 95% confidence intervals. 95% confidence intervals were calculated using Mid- P exact test. All analyses were performed with R version 3.3.2 [21].

## Results

A total of 6975 women were identified as having been diagnosed with ovarian cancer between 1990 and 2014 in BC. After excluding women who did not have EOC (*n* = 407), and after excluding women who were not captured in the death registry and were not registered for health care in BC in 2014 or for at least 5 years post diagnosis (*n* = 141), 6427 were included in our study. The study cohort included 2996 (46.6%) serous, 366 (5.7%) mucinous, 719 (11.2%) endometrioid, 431 (6.7%) clear cell and 1915 (29.8%) not classified EOCs.

The clinical characteristics of BC women diagnosed with EOC between 1990 and 2014 are outlined in Table 2. Among all histotypes, serous carcinomas were commonly observed (*n* = 2996), accounting for approximately 46.6% of total EOCs (66.4% if we remove EOCs that were not classified by histotype). The majority (68.2%, *n* = 4382) of affected women were diagnosed with EOC between 50 and 79 years of age and were not *BRCA* mutation carriers (96.9%, *n* = 6228).

**Table 2:** Clinical characteristics of the study cohort

Year of diagnosis	*N*	%
1990–1994	1106	17.2
1995–1999	1265	19.7
2000–2004	1252	19.5
2005–2009	1395	21.7
2010–2014	1409	21.9
Histology	N	%
Serous	2996	46.6
Mucinous	366	5.7
Endometrioid	719	11.2
Clear cell	431	6.7
Not classified	1915	29.8
Age at diagnosis	N	%
< 40	410	6.4
40–49	815	12.7
50–59	1477	23.0
60–69	1457	22.7
70–79	1448	22.5
80+	820	12.8
*BRCA* status	N	%
*BRCA* null	199	3.1
*BRCA* wild type	6228	96.9

Median follow- up was 3.5 years (IQR: 3.4–3.5 years) for women with serous EOCs, 9 years (IQR: 8–10 years) for women with mucinous EOCs, 7.5 years (IQR: 7–8.5 years) for women with endometrioid EOCs, 7 years (IQR: 6.5–8 years) for women with clear cell EOCs, and 4 years (IQR: 3.5–4 years) for women with not-classified EOCs.

By the end of this study, 55.9% of the study cohort (all histologies) died from their ovarian cancer (*n* = 3592), 33.9% were alive (*n* = 2181), 10.2% of women died from causes other than ovarian cancer (*n* = 654); 0.8% from breast cancer (*n* = 49), 0.5% from colorectal cancer (*n* = 34), 3.5% from ‘other’ cancers (*n* = 227), 1.8% from cardiovascular disease (*n* = 116), 1.8% from other chronic conditions (*n* = 114), 0.3% from external causes (*n* = 20) and 1.5% from unclassified causes (*n* = 94). Among women with serous EOCs, 29.3% (*n* = 877) were alive at the end of follow-up while 62.2% had died from their disease (*n* = 1864). 54.4% women with mucinous EOCs were alive (*n* = 199) while 32% died from ovarian cancer (*n* = 117). The greatest number of women alive at the end of follow-up was observed among women with endometrioid EOC (62.0%, *n* = 446). Among these women who had died, 26.7% died from ovarian cancer (*n* = 192). There was a considerable number of women with clear cell EOCs who were alive at the end of follow-up (55.9%, *n* = 241). However, 35.3% of women with clear cell EOCs died from ovarian cancer (*n* = 152). Among women with EOCs that could not be classified into histotypes, 21.8% of cases were alive at the end of follow-up (*n* = 418) while 66.2% died from ovarian cancer (*n* = 1267). Within each histotype, the most common cause of death after ovarian cancer was death from other cancers (Additional file 1: Table S3).

**Fig. 1:**
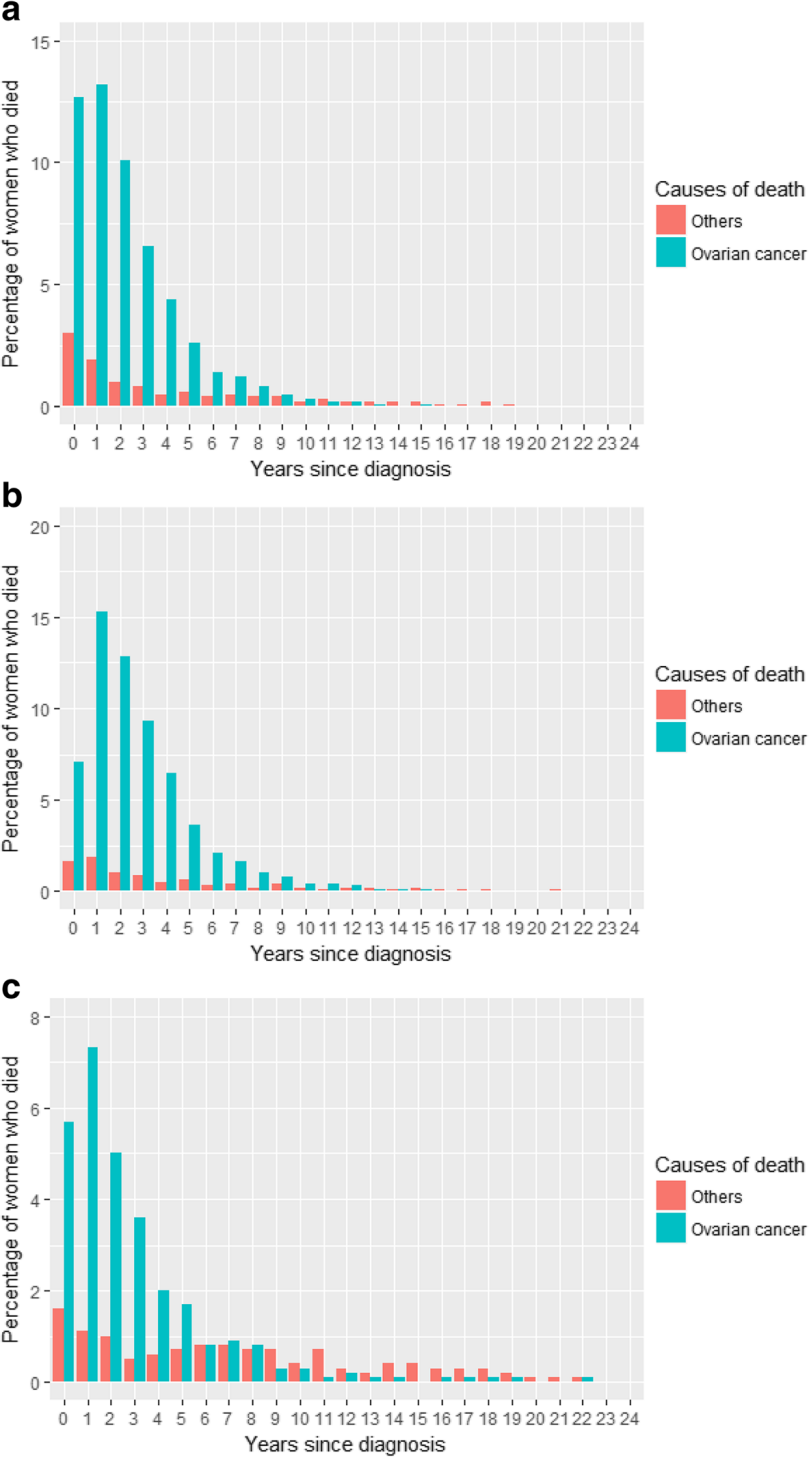
**a**: Frequency distribution of deaths among patients diagnosed with all the histotypes. **b**: Frequency distribution of deaths among patients diagnosed with serous epithelial ovarian cancers. **c**: Frequency distribution of deaths among patients diagnosed with non- serous epithelial ovarian cancers

**Fig. 2:**
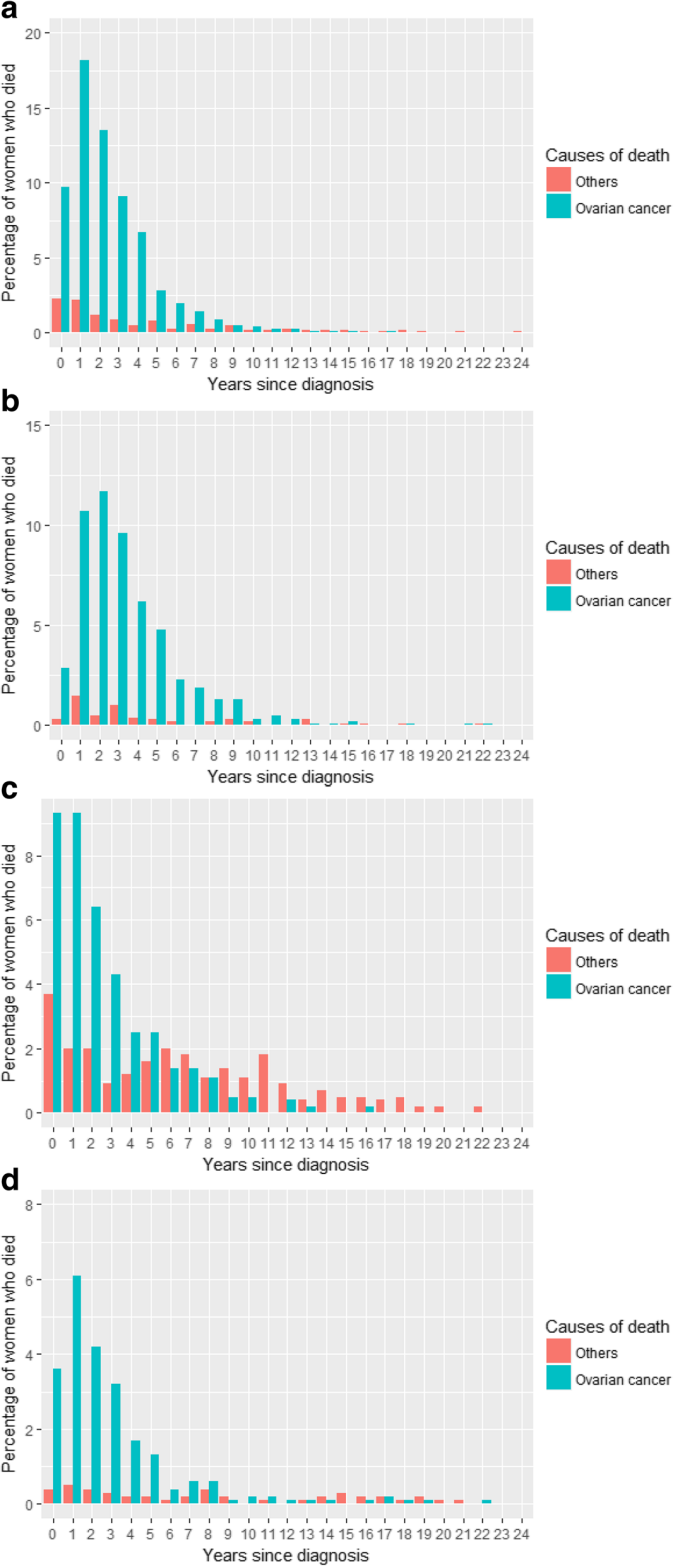
**a**: Frequency distribution of deaths among older patients (60 years or more) diagnosed with serous epithelial ovarian cancers. **b**: Frequency distribution of deaths among younger patients (under 60 years) diagnosed with serous epithelial ovarian cancers. **c**: Frequency distribution of deaths among older patients (60 years or more) diagnosed with non- serous epithelial ovarian cancers. **d**: Frequency distribution of deaths among younger patients (under 60 years) diagnosed with non- serous epithelial ovarian cancers

Figure 1a displays the frequency distribution of deaths for all histotypes. It reveals that ovarian cancer is the leading cause of death among women diagnosed with ovarian cancer for 10 years post diagnosis. It is first surpassed by other causes of deaths 11 years post diagnosis. Figure 1b displays the frequency distribution of deaths for serous EOCs. Ovarian cancer remains the leading cause of death among women diagnosed with serous EOCs for 12 years following diagnosis. Figure 1c depicts the frequency distribution of deaths for non- serous (endometrioid, clear cell, mucinous) EOCs. Other causes of death surpass ovarian cancer as the leading cause among women diagnosed with non-serous EOCs at 8 years post diagnosis. Figure 2a and b display the frequency distribution of deaths for serous EOCs by age group. Figure 2c and d displays the frequency distribution of deaths for non-serous EOCs by age group. Ovarian cancer remains the leading cause of death for longer among younger women (< 60 years) than among older women (60 years or more). Other causes of death first surpass ovarian cancer as the leading cause of death among older women with serous EOCs at 8 years post diagnosis. Whereas ovarian cancer is the leading cause of death among younger women with serous EOCs for 12 years post diagnosis. Among women with non- serous EOCs, ovarian cancer is the leading cause of death among older women for 5 years after diagnosis in comparison to younger women where it is 8 years after diagnosis. There were too few deaths from causes other than ovarian cancer in the women with a *BRCA* mutation to reliably examine causes of death over time in this group.

Differences between age groups in the causes of death, stratified by histotype, are reported in Additional file 2: Table S4. In each histotype group, a greater number of women who were diagnosed under 60 years of age survived than older women. Women diagnosed with serous cancer at 60 or older were most likely to die from ovarian cancer (66.8%, *n* = 1231), whereas women diagnosed with endometrioid cancer under the age of 60 were least likely to die from ovarian cancer (19.5%, *n* = 87) followed closely by women diagnosed with mucinous cancer under the age of 60 (21.3%, *n* = 46). For each age cohort, and for each histotype ovarian cancer was the leading cause of death. In all, death from ovarian cancer was most common, followed by ‘other’ cancers.

The outcomes of women with ovarian cancer and a *BRCA* mutation, stratified by serous or non- serous histotype is reported in Additional file 3: Table S5. We were unable to stratify by other histotypes due to small sample sizes. Among women diagnosed with serous cancer, those with a *BRCA* mutation (*BRCA* positive) face greater risk of death from breast cancer (4%, *n* = 6) in comparison to women without a mutation (0.7%, *n* = 20). In all, regardless of a woman’s *BRCA* mutation status, and for all histotypes ovarian cancer was the leading cause of death.

## Discussion

Herein we report that among EOC patients, ovarian cancer is the leading cause of death for 10 years post diagnosis before it is surpassed by other causes of death. However, this differed significantly based on histotype and age at diagnosis. For example, among women with serous EOC, ovarian cancer was the leading cause of death for 12 years compared to 8 years among women with non- serous EOCs. When stratified by age, ovarian cancer was the leading cause of death for 8 years post diagnosis among women with serous EOCs diagnosed after age 60 compared to 12 years among younger women (< 60 years of age) with serous EOCs. Our results suggest that women with non- serous EOCs were more likely to die from causes other than ovarian cancer in comparison to serous patients, as the majority of women with serous EOCs died from ovarian cancer. For instance, women with endometrioid and mucinous EOCs were more vulnerable to die from cardiovascular diseases, chronic conditions and unclassified causes than women with serous EOC.

Although the percentages of death from breast cancer were not significantly different across the histotypes, we found that these percentages varied when stratified by *BRCA* status. Women with a *BRCA* mutation and with serous carcinomas were less likely to die from other causes, and more likely to die from ovarian cancer, than women without a *BRCA* mutation. Whereas women with a *BRCA* mutation and a non- serous cancer were at relatively comparable risk of death from other causes as women without a *BRCA* mutation and a non- serous cancer.

Our results reporting a significant risk of death from ovarian cancer for many years post diagnosis is consistent with those previously reported using SEER data [5]. The SEER study reported that the probability of death from ovarian cancer decreases with increased survival years post diagnosis and the probability of death from all other causes increases. We observed that ovarian cancer is the leading cause of death among BC women diagnosed with EOC for 10 years post diagnosis. The SEER study reported that ovarian cancer was surpassed by other causes of death 7 years after diagnosis.

Our results are also consistent with a small body of literature reporting minimal risk of breast cancer among women with EOC and a *BRCA* mutation [22]. Our findings indicate a low incidence of death from breast cancer among women with ovarian cancer and a *BRCA* mutation. When stratified by histotype, *BRCA* mutation carriers diagnosed with serous EOCs were at the greater risk of death from breast cancer, breast cancer was only responsible for 4% of deaths among these women. This supports the assertion that there is no need to rush to perform mastectomy to prevent breast cancer among women with a *BRCA* mutation and EOC. Other research has suggested that there are greater improvements in survival with mastectomy among women who had already survived 10 years from diagnosis with EOC and those with stage I or II ovarian cancer. Mastectomy is also often recommended for ovarian cancer patients who are under age 55 and those with serum CA125 levels within normal limits [23, 24].

The population-based nature of the study and its inclusion of all women diagnosed with EOC in BC between 1990 and 2014 is an important strength of our research; however, some limitations are noted. Our reliance on the ICD morphologic codes to classify tumours into histologic subtypes likely introduced some misclassification. Although there have been considerable advances in categorization of epithelial ovarian cancer subtypes with high interobserver agreement in histotype assignment for this disease [25], our study includes cancers subtyped prior to these publications, and most did not have the benefit of additional immunohistochemical tests to help characterize challenging cases. Thus, we expect that there has been some misclassification of histotypes in this study. In addition, the rarity of ovarian cancer combined with the relatively small BC population has resulted in small numbers of women with histotypes other than serous ovarian cancer, and thus we had to group these histotypes in some analyses. Also, we cannot comment on the lynch syndrome status of our study population as we lack these data. Based on the past literature, women with endometroid and clear cell EOCs were observed to be at risk of Lynch syndrome [26]. Associated with a high risk of colorectal cancer, lynch syndrome status may have factored into the risk of death from colorectal cancer.

Importantly, we were unable to examine long-term mortality by stage because these data were missing in our cancer registry. Previous research has shown that women diagnosed with early stage EOC are less likely to die from ovarian cancer [5], but this has never been examined by histotype. Future research should examine whether stage influences long-term mortality differently by histotype. We are also missing data on important treatment variables, including the receipt of neoadjuvant or adjuvant chemotherapy, both intravenous and intraperitoneal, and the residual disease status of the study population following surgery. A better understanding of the effect of treatment by histotype would be useful for providing a more nuanced picture of what influences survival. Finally, our percentage of ovarian cancer patients with *BRCA* mutations is much lower than expected, given that recent research indicates approximately 20% of HGSC patients have a *BRCA* mutation [27]. This reflects the fact that histotype-based referral did not come into effect in British Columbia until 2010, and that even following this recommendation, only 46% of HGSC patients were being tested for *BRCA* mutations [28]. The high number of deaths from unclassified causes is unexpected and may reflect inaccuracies or missing information in the death certificates for some of those women. To limit this possible bias, our main analysis compared ovarian cancer specific mortality to all other causes of death.

The results of this study have implications for clinicians and ovarian cancer patients. The findings can help clinicians better understand the differences in outcomes among women with ovarian cancer based on histotype, age at diagnosis and *BRCA* status.

## Conclusions

Our data show that ovarian cancer remains the leading cause of death among women with EOC for 10 years post diagnosis. However, the probability of ovarian- cancer related death varies significantly based on histotype, age at diagnosis and *BRCA* mutation status. The differences in proportions of deaths between the histological groups support the evidence of ovarian cancer heterogeneity and support the assertion that histotype-specific research is valuable when researching ovarian cancer.

## Additional files

Additional file 1: **Table S3.** Cause of death stratified by histotype. (DOCX 18 kb)

Additional file 2: **Table S4.** Cause of death stratified by histotype and age at diagnosis. (DOCX 19 kb)

Additional file 3: **Table S5.** Cause of death stratified by histotype and *BRCA* status. (DOCX 16 kb)

## Abbreviations

BC: British Columbia
EOC: Epithelial ovarian cancer
HCP: BC hereditary cancer program
ICD-10: International Statistical Classification of Diseases codes: tenth revision (ICD-10)
SEER: Surveillance Epidemiology, and End Results
UCOD: Underlying cause of death
