# Supplementary material for: Long-term mortality among women with epithelial ovarian cancer: a population-based study in British Columbia, Canada
Source: BMC Cancer. 2018 Oct 25;18:1039. doi: 10.1186/s12885-018-4970-9 (PMC6202883; doi:10.1186/s12885-018-4970-9)
Supplement: Supplementary file 1 — Table S3. Cause of death stratified by histotype. (DOCX 18 kb) [file 12885_2018_4970_MOESM1_ESM.docx]

Table 3 Cause of death stratified by histotype

| Cause of death, N (%; 95% CI) | All EOC patients  (n= 6427) | Serous  (n=2996) | Endometrioid  (n = 719) | Clear cell  (n= 431) | Mucinous  (n= 366) | Not classified  (n= 1915) |
| --- | --- | --- | --- | --- | --- | --- |
| Alive | 2181  (33.9; 32.8, 35.1) | 877  (29.3; 27.7, 30.9) | 446  (62.0; 58.4, 65.5) | 241  (55.9; 51.2, 60.5) | 199  (54.4; 49.3, 59.4) | 418  (21.8; 20.0, 23.7) |
| Ovarian cancer | 3592 (55.9; 54.7, 57.1) | 1864 (62.2; 60.5, 63.9) | 192  (26.7; 23.6, 30.1) | 152  (35.3; 30.9, 39.9) | 117  (32; 27.4, 36.9) | 1267  (66.2; 64.0, 68.2) |
| Breast cancer | 49  (0.8; 0.6, 1) | 26 (0.9; 0.6, 1.3) | 5  (0.7; 0.3, 1.6) | -- | -- | 13  (0.7; 0.4, 1.2) |
| Colorectal cancer | 34 (0.5; 0.6, 0.7) | 14  (0.5; 0.3, 0.8) | 6  (0.8; 0.4, 1.8) | -- | -- | 11  (0.6; 0.3, 1) |
| Other cancer | 227 (3.5; 3.1, 4.0) | 82 (2.7; 2.2, 3.4) | 19  (2.6; 1.7, 4.1) | 9  (2.1; 1.1, 3.9) | 19  (5.2; 3.4, 8) | 98  (5.1; 4.2, 6.2) |
| Cardiovascular disease | 116 (1.8; 1.5, 2.2) | 46  (1.5; 1.2, 2.1) | 18  (2.5; 1.6, 3.9) | -- | 11 (3.0; 1.7, 5.3) | 37  (1.9; 1.4, 2.7) |
| Other chronic | 114  (1.8; 1.5, 2.1) | 37  (1.2; 0.9, 1.7) | 17  (2.4; 1.5, 3.8) | 8  (1.9; 1, 3.6) | 11  (3.0; 1.7, 5.3) | 41  (2.1; 1.6, 2.9) |
| External causes | 20  (0.3; 0.2, 0.5) | 12  (0.4; 0.2, 0.7) | -- | -- | -- | -- |
| Unclassified causes | 94  (1.5; 1.2, 1.8) | 38  (1.3; 0.9, 1.7) | 14  (2; 1.2, 3.3) | 8  (1.9; 1, 3.6) | 5 (1.4; 0.6, 3.2) | 29  (1.5; 1.1, 2.2) |

-- suppressed due to small cell sizes
