# Supplementary material for: Long-term mortality among women with epithelial ovarian cancer: a population-based study in British Columbia, Canada
Source: BMC Cancer. 2018 Oct 25;18:1039. doi: 10.1186/s12885-018-4970-9 (PMC6202883; doi:10.1186/s12885-018-4970-9)
Supplement: Supplementary file 2 — Table S4. Cause of death stratified by histotype and age at diagnosis. (DOCX 19 kb) [file 12885_2018_4970_MOESM2_ESM.docx]

Table 4 Cause of death stratified by histotype and age at diagnosis

| Cause of death, N (%; 95% CI) | Serous  (n=2996) | | Endometrioid  (n = 719) | | Clear cell  (n= 431) | | Mucinous  (n= 366) | | Not classified  (n= 1915) | |
| --- | --- | --- | --- | --- | --- | --- | --- | --- | --- | --- |
|  | <60 (n= 1153) | >=60  (n= 1843) | <60 (n= 446) | >=60  (n= 273) | <60 (n= 293) | >=60  (n=138) | <60 (n= 216) | >=60  (n=150) | <60 (n= 594) | >=60  (n= 1321) |
| Alive | 462 (40.0;  32.3, 42.9) | 415 (22.5;  20.7, 24.5) | 340  (76.2;  72.1, 79.9) | 106  (38.3;  33.2, 44.7) | 191  (65.2;  59.6, 70.4) | 50  (36.2; 28.7, 44.5) | 158  (73.1;  66.9, 78.6) | 41  (27.3; 20.8,34.9) | 299  (50.3;  46.3, 54.4) | 119  (9;  7.6, 10.7) |
| Ovarian cancer | 633  (54.9;  52, 57.8) | 1231 (66.8;  64.6, 68.9) | 87 (19.5;  16.1, 23.4) | 105  (38.5;  32.9, 44.4) | 92  (31.4;  26.4, 36.9) | 60  (43.5;  35.5, 51.8) | 46  (21.3;  16.4, 27.2) | 71  (47.3;  39.5,  55.3) | 253  (42.6; 38.7, 46.6) | 1014  (76.8;  74.4, 79) |
| Breast cancer | 13 (1.1;  0.7, 1.9) | 13 (0.7;  0.4, 1.2) | -- | -- | -- | -- | -- | -- | -- | 9  (0.7;  0.4, 1.3) |
| Colorectal cancer | -- | 12  (0.7;  0.4, 1.1) | -- | -- | -- | -- | -- | -- | -- | 8  (0.6;  0.3, 1.2) |
| Other cancer | 15  (1.3;  0.8, 2.1) | 67  (3.6;  2.9, 4.6) | 6 (1.4;  0.6, 2.9) | 13  (4.8;  2.8, 8) | -- | 8  (5.8;  3, 11) | 7  (3.2;  1.6, 6.5) | 12  (8;  4.6, 13.5) | 22  (3.7;  2.5, 5.5) | 76  (5.8;  4.6, 7.1) |
| Cardiovascular disease | 8  (0.7;  0.4, 1.4) | 38  (2.1;  1.5, 2.8) | -- | 17 (6.2;  3.9, 9.8) | -- | -- | -- | 8  (5.3;  2.7, 10.2) | -- | 35  (2.7;  1.9, 3.7) |
| Other chronic | 11  (1.0;  0.5, 1.7) | 26  (1.4; 1,  2.1) | -- | 13  (4.8;  2.8, 8) | -- | 5  (0.3;  0.1, 0.8) | -- | 11  (7.3;  4.1, 12.7) | 5  (0.8;  0.4, 2) | 36  (2.7;  2, 3.8) |
| External causes | -- | 9 (0.5;  0.3, 0.9) | -- | -- | -- | -- | -- | -- | -- | -- |
| Unclassified causes | 6  (0.5;  0.2, 1.1) | 32 (1.7;  1.2, 2.5) | -- | 12  (4.4;  2.5, 7.5) | -- | 8  (5.8;  3, 11.0) | -- | 5  (3.3;  1.4, 7.6) | 6  (1.0;  0.5, 2.2) | 23  (1.7;  1.2, 2.6) |

-- suppressed due to small cell sizes
