# Supplementary material for: Long-term mortality among women with epithelial ovarian cancer: a population-based study in British Columbia, Canada
Source: BMC Cancer. 2018 Oct 25;18:1039. doi: 10.1186/s12885-018-4970-9 (PMC6202883; doi:10.1186/s12885-018-4970-9)
Supplement: Supplementary file 3 — Table S5. Cause of death stratified by histotype and BRCA status. (DOCX 16 kb) [file 12885_2018_4970_MOESM3_ESM.docx]

Table 5 Cause of death stratified by histotype and *BRCA* status

| Cause of death, N (%; 95% CI) | Serous  (n=2996) | | Non- serous  (n = 1516) | |
| --- | --- | --- | --- | --- |
|  | BRCA negative (n= 2846) | BRCA positive  (n= 150) | BRCA negative (n= 1502) | BRCA positive  (n= 14) |
| Alive | 798  (28.0; 26.4, 29.7) | 79  (52.7; 44.7, 60.5) | 879  (58.5; 56.0, 60.1) | 7  (50; 26.8, 73.2) |
| Ovarian cancer | 1802  (63.3; 61.5, 65.1) | 62  (41.3; 33.8, 49.3) | 455  (30.3; 28.0, 32.7) | -- |
| Breast cancer | 20  (0.7; 0.5, 1.1) | 6  (4; 1.8, 8.4) | 10  (0.7; 0.4, 1.2) | -- |
| Other causes | 214  (7.5; 6.6, 8.6) | -- | 151  (10.1; 8.6, 11.7) | 6  (42.9; 21.4, 67.4) |

-- suppressed due to small cell sizes
